# Supplementary material for: Iterative improvement in the automatic modular design of robot swarms
Source: PeerJ Comput Sci. 2020 Dec 7;6:e322. doi: 10.7717/peerj-cs.322 (PMC7924708; doi:10.7717/peerj-cs.322)
Supplement: Supplemental Information 3 [file peerj-cs-06-322-s003.zip › argos3/doc/api/standalone/a00611_source.html]

ARGoS: plugins/simulator/physics\_engines/dynamics2d/dynamics2d\_multi\_body\_object\_model.cpp Source File


- Main Page
- Related Pages
- Namespaces
- Classes
- Files

- File List
- File Members

# plugins/simulator/physics\_engines/dynamics2d/dynamics2d\_multi\_body\_object\_model.cpp

Go to the documentation of this file.

```
00001 #include "dynamics2d_multi_body_object_model.h"
00002 #include <argos3/core/simulator/entity/composable_entity.h>
00003 
00004 namespace argos {
00005 
00006    /****************************************/
00007    /****************************************/
00008 
00009    CDynamics2DMultiBodyObjectModel::CDynamics2DMultiBodyObjectModel(CDynamics2DEngine& c_engine,
00010                                                                     CComposableEntity& c_entity) :
00011       CDynamics2DModel(c_engine, c_entity.GetComponent<CEmbodiedEntity>("body")),
00012       m_cEntity(c_entity) {}
00013 
00014    /****************************************/
00015    /****************************************/
00016 
00017    CDynamics2DMultiBodyObjectModel::~CDynamics2DMultiBodyObjectModel() {
00018       /* Dispose of shapes and bodies */
00019       while(!m_vecBodies.empty()) {
00020          /* Get pointer to body */
00021          cpBody* ptBody = m_vecBodies.back().Body;
00022          /* Remove all of its shapes */
00023          cpShape* ptCurShape = ptBody->shapeList;
00024          cpShape* ptNextShape;
00025          while(ptCurShape) {
00026             ptNextShape = ptCurShape->next;
00027             cpSpaceRemoveShape(GetDynamics2DEngine().GetPhysicsSpace(), ptCurShape);
00028             cpShapeFree(ptCurShape);
00029             ptCurShape = ptNextShape;
00030          }
00031          /* Dispose of body */
00032          cpSpaceRemoveBody(GetDynamics2DEngine().GetPhysicsSpace(), ptBody);
00033          cpBodyFree(ptBody);
00034          /* Dispose of struct */
00035          m_vecBodies.pop_back();
00036       }
00037    }
00038 
00039    /****************************************/
00040    /****************************************/
00041 
00042    void CDynamics2DMultiBodyObjectModel::MoveTo(const CVector3& c_position,
00043                                                 const CQuaternion& c_orientation) {
00044       /* Set target position and orientation */
00045       cpVect tBodyPos = cpv(c_position.GetX(), c_position.GetY());
00046       CRadians cXAngle, cYAngle, cZAngle;
00047       c_orientation.ToEulerAngles(cZAngle, cYAngle, cXAngle);
00048       cpFloat tBodyOrient = cZAngle.GetValue();
00049       /* For each body: */
00050       for(size_t i = 0; i < m_vecBodies.size(); ++i) {
00051          /* Set body orientation at anchor */
00052          cpBodySetAngle(m_vecBodies[i].Body,
00053                         tBodyOrient + m_vecBodies[i].OffsetOrient);
00054          /* Set body position at anchor */
00055          cpBodySetPos(m_vecBodies[i].Body,
00056                       cpvadd(tBodyPos,
00057                              cpvrotate(m_vecBodies[i].OffsetPos,
00058                                        m_vecBodies[i].Body->rot)));
00059          
00060          /* Update shape index */
00061          cpSpaceReindexShapesForBody(GetDynamics2DEngine().GetPhysicsSpace(),
00062                                      m_vecBodies[i].Body);
00063       }
00064       /* Update ARGoS entity state */
00065       UpdateEntityStatus();
00066    }
00067 
00068    /****************************************/
00069    /****************************************/
00070 
00071    void CDynamics2DMultiBodyObjectModel::Reset() {
00072       /* Reset body position */
00073       MoveTo(GetEmbodiedEntity().GetOriginAnchor().Position,
00074              GetEmbodiedEntity().GetOriginAnchor().Orientation);
00075       /* For each body: */
00076       for(size_t i = 0; i < m_vecBodies.size(); ++i) {
00077          /* Zero the speeds */
00078          m_vecBodies[i].Body->v = cpvzero;
00079          m_vecBodies[i].Body->w = 0.0f;
00080          /* Zero forces and torques */
00081          cpBodyResetForces(m_vecBodies[i].Body);
00082       }
00083    }
00084 
00085    /****************************************/
00086    /****************************************/
00087 
00088    void CDynamics2DMultiBodyObjectModel::CalculateBoundingBox() {
00089       if(m_vecBodies.empty()) return;
00090       cpBB tBoundingBox;
00091       Real fMaxHeight;
00092       for(size_t i = 0; i < m_vecBodies.size(); ++i) {
00093          tBoundingBox = cpShapeGetBB(m_vecBodies[i].Body->shapeList);
00094          for(cpShape* pt_shape = m_vecBodies[i].Body->shapeList->next;
00095              pt_shape != NULL;
00096              pt_shape = pt_shape->next) {
00097             cpBB* ptBB = &pt_shape->bb;
00098             if(ptBB->l < tBoundingBox.l) tBoundingBox.l = ptBB->l;
00099             if(ptBB->b < tBoundingBox.b) tBoundingBox.b = ptBB->b;
00100             if(ptBB->r > tBoundingBox.r) tBoundingBox.r = ptBB->r;
00101             if(ptBB->t > tBoundingBox.t) tBoundingBox.t = ptBB->t;
00102          }
00103          fMaxHeight = Max(fMaxHeight, m_vecBodies[i].Height);
00104       }      
00105       GetBoundingBox().MinCorner.SetX(tBoundingBox.l);
00106       GetBoundingBox().MinCorner.SetY(tBoundingBox.b);
00107       GetBoundingBox().MinCorner.SetZ(GetDynamics2DEngine().GetElevation());
00108       GetBoundingBox().MaxCorner.SetX(tBoundingBox.r);
00109       GetBoundingBox().MaxCorner.SetY(tBoundingBox.t);
00110       GetBoundingBox().MaxCorner.SetZ(GetDynamics2DEngine().GetElevation() + fMaxHeight);
00111    }
00112 
00113    /****************************************/
00114    /****************************************/
00115 
00116    bool CDynamics2DMultiBodyObjectModel::IsCollidingWithSomething() const {
00117       if(m_vecBodies.empty()) return false;
00118       for(size_t i = 0; i < m_vecBodies.size(); ++i) {
00119          for(cpShape* pt_shape = m_vecBodies[i].Body->shapeList;
00120              pt_shape != NULL;
00121              pt_shape = pt_shape->next) {
00122             if(cpSpaceShapeQuery(
00123                   const_cast<CDynamics2DMultiBodyObjectModel*>(this)->
00124                   GetDynamics2DEngine().GetPhysicsSpace(),
00125                   pt_shape, NULL, NULL) > 0) {
00126                return true;
00127             }
00128          }
00129       }
00130       return false;
00131    }
00132 
00133    /****************************************/
00134    /****************************************/
00135 
00136    void CDynamics2DMultiBodyObjectModel::AddBody(cpBody* pt_body,
00137                                                  const cpVect& t_offset_pos,
00138                                                  cpFloat t_offset_orient,
00139                                                  Real f_height) {
00140       /* Set the body and its data field for ray queries */
00141       pt_body->data = this;
00142       /* Add body to list */
00143       m_vecBodies.push_back(SBody(pt_body,
00144                                   t_offset_pos,
00145                                   t_offset_orient,
00146                                   f_height));
00147       /* Calculate the bounding box */
00148       CalculateBoundingBox();
00149    }
00150 
00151    /****************************************/
00152    /****************************************/
00153 
00154    CDynamics2DMultiBodyObjectModel::SBody::SBody(cpBody* pt_body,
00155                                                  const cpVect& t_offset_pos,
00156                                                  cpFloat t_offset_orient,
00157                                                  Real f_height) :
00158       Body(pt_body),
00159       OffsetPos(t_offset_pos),
00160       OffsetOrient(t_offset_orient),
00161       Height(f_height) {}
00162 
00163    /****************************************/
00164    /****************************************/
00165 
00166 }
```

---

Generated on 10 Jul 2018 for ARGoS by 
 1.6.1
